# The Inseparable Three: How Organization and Culture Can Foster Individual Creativity

**DOI:** 10.3389/fpsyg.2019.02133

**Published:** 2019-09-18

**Authors:** Yoannis Hermida, Willow Clem, C. Dominik Güss

**Affiliations:** Department of Psychology, University of North Florida, Jacksonville, FL, United States

**Keywords:** culture, cross-cultural, organization, creativity, cultural values, innovation

## Introduction and Overview

Creativity can be defined as coming up with novel and useful ideas or products (Guilford, 1950; Kim and Zhong, 2017). Although past research has shed light on the various factors that influence creativity, few studies have examined influences on creativity with respect to the individual, organization, and culture, or interactions among these influences. Even though there is a lack of research in this field, it is a highly important topic for international companies with employees from various cultural and ethnic backgrounds working together in order to maximize their potential for innovation and success in competitive markets (Pitta et al., 2008; Fischer et al., 2016). In this article, we will discuss recent work on individual, organizational, and cultural processes that foster and suppress creativity. Our discussion is by no means exhaustive, but focuses on conditions that can bring out the creative potential of employees.

We will discuss how an individual's creativity can be stimulated through forward flow, personal need for structure, and proactive personality characteristics. Organizational structures that foster an individual's creativity include a strong support system, transfer of knowledge, and management styles. Finally, cultural structures that encourage an individual's creativity comprise of multicultural interactions, individualistic and collectivist cultural values, and cultural looseness. To conclude, we integrate the findings and end with suggestions for future research in this field.

## Individual and Creativity

First, a new metric term, “forward flow” (FF), is used to quantify free association (Gray et al., 2019). Whereas, creativity has often been defined as divergent thinking, i.e., thinking in different directions, FF is defined by Gray et al. (2019) as the extent to which present thoughts deviate from past thoughts within free association. The authors used a latent semantic analysis to capture the semantic transformation of thoughts over time. Gray et al. (2019) investigated whether FF could predict creativity. Indeed, in several studies involving college students, a representative sample of Americans, professional actors, and entrepreneurs, FF predicted creativity. The results of this research suggest that creativity can be stimulated by instructing people to (a) think in different directions and (b) continue thinking along these lines, i.e., think broadly and think deeply into the future.

Second, personal need for structure is another individual variable that holds the potential to hinder or augment creativity. Past research has shown that personal need for structure (PNS) is negatively associated with creative performance (Thompson et al., 2001). However, Rietzschel et al. (2007) investigated a moderating variable that could potentially affect this relationship—personal fear of invalidity (PFI). If PFI is high, PNS should be inversely related to creativity. Conversely, if PFI is low, PNS should be positively related to creativity, as it allows individuals to tackle a creative task using a structured method without fear of invalidity. Results of the four studies confirmed these hypotheses for creative fluency and originality (Rietzschel et al., 2007). These findings show the interrelationship between the individual and social component of creativity. PFI can be influenced through leadership and organizational climate. If organizations provide a “supportive” environment, in which employees do not need to feel fearful of criticism and consequences, employees who experience a personal need for structure can express their creative potential and become more proactive.

Employee innovativeness is influenced by proactive personalities who are highly engaged in the daily challenges involved at work (Salanova et al., 2005). Proactive personalities are likely to participate in generating, distributing, and executing novel ideas as they consistently look for methods to refine their current circumstances, all of which are positively related to innovation (Crant, 2000; Li et al., 2017). In addition, proactive personalities are inclined to engage in positive affect in the workplace and report stronger work engagement—working more hours per week while eagerly encouraging the accomplishment of original ideas (Kong and Li, 2018).

## Organization and Creativity

Creativity in the workplace is regarded as the main cause for business and organizational success and a company's competitiveness (Liu, 2018). It is, therefore, no surprise that research has focused on investigating creativity in the workplace and its contributing factors. Previous research shows that workforce diversity can enhance individual and group creativity compared to less diverse organizations (Han et al., 2011; Hoever et al., 2012) which ultimately leads to creativity in an organization. Current research supports these findings, where Luu (2019) found that maintaining a diverse group of individuals within an organization fostered service innovativeness among employees in Brazilian and Vietnamese companies.

Creating a climate conducive for creativity also involves supportive leadership (Chow, 2018). While general models of business leadership have drawn substantially from studies of American companies, one study (Cappelli et al., 2015) has identified a unique leadership concept within Indian companies: broad mission and purpose. Many Indian business leaders place importance not only on stockholder demands but also on family prosperity, regional progression, and national expansion. Indian business executives also make greater use of transformational management (i.e., focusing on overarching goals of the organization) rather than transactional management (i.e., striking deals with subordinates to reach a solution). The differences found between Western and Indian business organizations lie in the extent to which their goals extend beyond financial considerations.

The atmosphere in the workplace, which primarily stems from the kind of leadership provided, also greatly influences employee's level of innovative productivity. Isaksen and Akkermans (2011) examined the influence of the workplace climate on creativity and innovation in over 100 organizations from 10 countries. Individuals who perceived greater leadership support for creativity showed higher scores in creative climates. Conveying a perceived interest in employees at a personal level can lead to high creative performance ratings (Kirkman and Rosen, 1997). Similarly, current research indicates that empowering leaders who motivate their subordinates by constructing an environment that supports, provides feedback, and offers resources and opportunities, enhances both efficacy and efficiency (Chow, 2018). These findings suggest that both leadership behavior and perceived support for innovative productivity foster workplace creativity. Thus, organizational climate matters.

## Culture and Creativity

Culture is another important influence on individual creativity. We previously mentioned this in our discussion of leadership preferences of Indian managers. Culture can be defined as acquired knowledge, functional in a specific environment and shared by a specific group of people to successfully cope with the environment and with each other (see e.g., Güss et al., 2010).

One variable related to creativity is multicultural experiences, experiences in another cultural environment or with people from another culture. Multicultural experiences (MCE) are now easier to access and attain through the advancement of video technology, the internet, and the ease of traveling. Research has shown a positive relationship between MCE and creativity (e.g., Leung et al., 2008). One study (Aytug et al., 2018) distinguished between two kinds of MCE—multicultural interaction and multicultural exposure. Multicultural interactions rather than multicultural exposures were significantly correlated with creativity (Aytug et al., 2018). These results indicate that experiencing cultural differences and processing differing perspectives through social interactions rather than superficial encounters with other cultures widen an individual's knowledge and enhance creativity (Gocłowska and Crisp, 2014).

Cultural tightness and looseness are also an important influence on creativity. Previous notions of successful innovation were thought to be dependent upon cultural alignment (i.e., cultural agreement) between the intended product and the audience's interpretation of what is acceptable (De Dreu, 2010). Chua et al. (2015) theorized that creativity in the workplace is contingent on cultural tightness (i.e., strong social norms and possessing a low tolerance for deviant behavior) as well as the cultural distance between the innovator's country and the audience's country. The results revealed that individuals from countries with tight cultural norms were less likely than those from countries with loose cultural norms to successfully engage and succeed in foreign creative tasks (e.g., culture-specific marketing). However, it should be noted that members of a tight culture were still able to perform creatively only if they worked with individuals from their own or culturally close countries (Chua et al., 2015). These results suggest that culturally loose countries have an advantage when dealing creatively with novel tasks in the global market compared to members of culturally tight countries (Chua et al., 2015).

Finally, research has shown an association between innovation and individualistic as opposed to collectivistic values (e.g., Goncalo and Staw, 2006). Past research finds that collectivistic environments stimulate cooperation and productivity while individualistic environments promote opportunism and hostile behavior (Locke et al., 2001). However, a recent study shows that integrating individualistic and collectivistic values ensures the enhancement of high levels of creativity (Bechtoldt et al., 2012). This study replicated Goncalo and Staw's (2006) research and additionally controlled for self-construal and value orientation in brainstorming tasks. Bechtoldt et al. (2012) found that the collectivistic value group produced more ideas than the individualistic value group. However, ideas were found to be more original when group members combined a collectivistic value orientation (i.e., high group motivation) and individualistic self-construal (i.e., high self-reliance). Thus, these findings demonstrate that incorporating elements from both individualistic and collectivistic values and norms can enhance originality. Other studies have shown that individualistic and collectivist values are a continuum, can coexist, and can influence cognitive decision-making strategies and performance in uncertain situations (e.g., Güss et al., 2010).

## The Inseparable Triad: Individual, Organization, and Culture

So far, we have discussed individual, organizational, and cultural influences on creativity separately. However, they cannot be separated and must be considered together as the following study shows. A proactive personality [i.e., a personality trait where individuals make things happen rather than waiting for them to happen (Grant and Ashford, 2008)] is a central dispositional factor in individuals' creativity (Sears et al., 2018). In two large studies of Chinese and Canadian participants, the authors found that proactive personality fosters workplace creativity through the two mediators of intrinsic motivation and interactional justice. Intrinsic motivation refers to an interest in a topic or activity for its own sake (Amabile and Pratt, 2016). Interactional justice refers to the sensitivity and respect supervisors show toward their employees' needs. The link between proactive personality, interactional justice, and creativity was, however, moderated by power distance: it was only significant for employees with a low level of power distance. It was additionally moderated by the supervisor having a proactive personality. Power distance is defined as accepting inequality in social hierarchies (Hofstede, 2001). These results show the interrelatedness of individual (cognitive-motivational), organizational (interactional justice) and cultural variables (power distance values) and the need to investigate variables and processes on the three levels together (see also Farh et al., 2007).

Other studies have highlighted the role of leadership in fostering employees' creativity. Formal visionary leaders—or transformational management styles—effectively provide guidance and communication to informal leaders or subordinates who possess proactive personalities which in turn, allow them to transform and utilize the necessary resources in order to tackle challenging situations and create new ideas (Yukl et al., 2002; Pan and Xu, 2018). Simply strengthening creative idea production, however, is not enough. Idea generation and idea execution go hand-in-hand in the creative process (Detert and Trevino, 2010). The cultural-value differences between management and employees can lead to employees' personal fear of invalidity when implementing their creative ideas (Kirkman and Chen, 2009; Taha et al., 2016; Pan and Xu, 2018). Pan and Xu (2018) noted that in tight, collectivist countries like China—where loyalty and commitment are highly regarded—employees must consider their manager's response to new ideas and therefore possess higher personal fear of invalidity. One might argue that combining individualism and collectivism allows for leaders to have the best of both worlds; creating a positive social environment conducive of creative ideas with constructive criticism, unencumbered by any fears of appearing wrong or incapable.

## Conclusion and Lessons

In our short review, we have shown that employees' creativity can be fostered by specific individual, organizational, and cultural factors. These findings have implications for management and organizational psychology; creating a strategy where multicultural organizations can identify and foster the factors that cultivate creativity at the individual, cultural, and organizational levels is essential. Individual traits that help foster creativity are forward flow, proactive personality, low fear of invalidity, and intrinsic motivation in creative tasks. On the organizational level, using transformational management styles and implementing a strong support system within the organization not only foster creativity but also secure the company's longevity and competitiveness. Lastly, regarding culture, creativity can be stimulated through interactions with people from different cultures, rather than superficial exposure. Additionally, cultural values can promote creativity: Combining collectivist value orientations (i.e., prioritizing the greater good of the group) with individualistic self-construal (i.e., defining oneself as independent), having low power distance, and, in novel tasks, cultural looseness.

We have discussed several studies that investigate either one aspect or several aspects of the creativity-triad: individual, organization, and culture. The following are some suggestions for future research in this area.

### Lesson 1: The Construct

Creativity is a complex and multifaceted construct. Future studies could further investigate empirically implicit theories on creativity in organizations across cultures. If there is congruence on major aspects of creativity, such as novelty and appropriateness, then cross-cultural comparisons are warranted. Before researchers discuss cultural differences, great care has to be taken to guarantee measurement equivalence, structural equivalence, and full scale equivalence (e.g., van de Vijver and Tanzer, 2004). Another way to investigate the construct is to use several measurements (see also Batey and Furnham, 2006) in order to assess convergent and discriminant validity.

Additionally, several researchers argue against a static view and investigation of innovation and creativity and argue for an investigation of dynamic cognitive processes (e.g., Cornelissen and Clarke, 2010). For a marketing specialist or a product designer team, creativity is a process that can take weeks, if not months or years. Thus, future research could address the dynamics of the creative process in organizations over time while looking at cultural differences.

### Lesson 2: The Samples

Several problems relate to samples in cross-cultural studies. First, many studies have focused on between-country comparisons potentially neglecting within-country differences. Potential differences among countries might be smaller than within-country differences among different samples. Second, many cross-cultural studies have often overgeneralized findings between a few Asian countries and the United States. Researchers might overgeneralize among Asian countries–speaking of East versus West–and minimize between-country differences in the West or between-country differences in the East. Thus, future research should not only focus on cross-cultural, but also on within-country comparisons of several countries from different continents—and not only on the US and one East Asian country (see also Tsai, 2012).

### Lesson 3: Method and Data Analysis

Qualitative methods, such as in-depth interviews (e.g., Güss et al., 2018), could also be utilized in cross-cultural research on creativity in organizations. Such data would reveal important themes in creativity and show the complexity of the process.

The creativity-triad individual-organization-culture could also be studied quantitatively with data analysis techniques that combine several variables and their interrelations, such as structural equation modeling. A theoretical path model, which states how cultural, organizational, and individual factors influence creativity, could be tested overall and for each country including multiple group analyses (for a similar procedure testing the influence of cultural values on decision making, see Güss, 2011). One step further would be hierarchical linear modeling or multilevel analyses with large data sets. Results would highlight the relative contribution of each factor (individual, organization, and culture) in explaining variance in creativity scores.

In sum, characteristics of individuals, organizations, and cultures interactively influence creativity and have to be regarded as having a multiplicative rather than additive relationship. When one of them is 0, the others become 0. Only looking at one of the three in isolation is like looking at one side of a triangle. It is exactly their interaction that creates a fertile foundation in which creative ideas and products can grow.

## Author Contributions

All authors listed have made a substantial, direct and intellectual contribution to the work, and approved it for publication.

### Conflict of Interest Statement

The authors declare that the research was conducted in the absence of any commercial or financial relationships that could be construed as a potential conflict of interest.
